# Supplementary material for: Complexome profiling of the Chlamydomonas psb28 mutant reveals TEF5 as an early PSII assembly factor
Source: Plant Cell. 2025 Mar 17;37(6):koaf055. doi: 10.1093/plcell/koaf055 (PMC12164590; doi:10.1093/plcell/koaf055)
Supplement: koaf055_Supplementary_Data [file koaf055_supplementary_data.zip › psb28_interactive.zip!/plots/intensity/qualityPlots/peptides/RBD1_Cre07.g315150.t1.2.html]

### Cre07.g315150.t1.2

Trivial name: RBD1  
SeqCoverage [median]: 14.80  
Mass: 18.17 kDa  
  
 

| Sequence | MissedCleavage | Mass [Da] | wtIntensity | muIntensity | Score |
| --- | --- | --- | --- | --- | --- |
| VKVVAGFAENQQYGLGGNSMTEAQK | 1 | 2625.29 | 0.00 | 628.26 | 55.53 |
| VVAGFAENQQYGLGGNSMTEAQK | 0 | 2398.12 | 876.14 | 12791.20 | 172.69 |
|  |  |  | 876.1 | 13419.5 |  |
